# The Mediating Role of Rumination in the Relation between Basic Psychological Need Frustration and Depressive Symptoms

**DOI:** 10.3390/jcm12020395

**Published:** 2023-01-04

**Authors:** Andreas Heissel, Anou Pietrek, Maria Kangas, Jolene Van der Kaap-Deeder, Michael A. Rapp

**Affiliations:** 1 Social and Preventive Medicine, Department of Sports and Health Science, Intra-Faculty Unit “Cognitive Sciences”, Faculty of Human Science, and Faculty of Health Sciences Brandenburg, Research Area Services Research and e-Health, University of Potsdam, 14469 Potsdam, Germany; 2 Social and Preventive Medicine, Department of Sports and Health Sciences, Faculty of Human Science, University of Potsdam, 14469 Potsdam, Germany; 3 Centre for Emotional Health, School of Psychological Sciences, Macquarie University, Sydney, NSW 2109, Australia; 4 Department of Psychology, Norwegian University of Science and Technology, 7034 Trondheim, Norway

**Keywords:** psychopathology, self-determination theory, response styles theory, frustration, depressive disorder, emotional regulation, rumination

## Abstract

Research within the framework of Basic Psychological Need Theory (BPNT) finds strong associations between basic need frustration and depressive symptoms. This study examined the role of rumination as an underlying mechanism in the association between basic psychological need frustration and depressive symptoms. A cross-sectional sample of N = 221 adults (55.2% female, mean age = 27.95, range = 18–62, SD = 10.51) completed measures assessing their level of basic psychological need frustration, rumination, and depressive symptoms. Correlational analyses and multiple mediation models were conducted. Brooding partially mediated the relation between need frustration and depressive symptoms. BPNT and Response Styles Theory are compatible and can further advance knowledge about depression vulnerabilities.

## 1. Introduction

Self-Determination Theory (SDT) is a broad theory on motivation, socialization, personality, and well-being. Within the framework of SDT, the mini-theory of Basic Psychological Needs Theory (BPNT) [1,2] provides a useful framework for understanding socio-environmental conditions that either support or thwart people’s basic psychological needs and their consequences. According to the BPNT [1,2], people have three inherent universal needs (i.e., autonomy, relatedness, and competence) that are important for personal psychological growth. Specifically, the need for autonomy refers to the need to experience one’s behaviour as self-initiated and reflecting one’s own interests; the need for competence reflects the need to experience mastery and capacities to achieve desired goals; the need for relatedness describes the need for experiencing connectedness and care in contact with significant others [1]. Some time ago, BPNT was extended to include the dimension of need frustration alongside need satisfaction [2,3,4], where the experience of need frustration cannot be equated with the absence of need satisfaction. To illustrate, not feeling close to one’s colleagues (low need satisfaction) is not the same as feeling actively excluded by these colleagues (need frustration). Thus, need frustration describes an experience of threat, or perceived reflection of hurt [5]. Under conditions of basic psychological need frustration, maladjustment, or even psychopathology are proposed to result [2,3,4]. Some studies have demonstrated the transdiagnostic nature of need frustration in psychopathology such as depressive symptoms, eating pathology and anxiety [6,7]. For depressive symptoms, a number of studies have demonstrated a strong positive association with need frustration [8,9,10,11,12]. For instance, in a sample of older adolescent athletes, Bartholomew et al. [8] found that need frustration significantly related to elevated depressive symptoms. Weinstein et al. [12] found that Syrian refugees who experienced more need frustration exhibited higher levels of depressive symptoms. Chen et al. [9] found that need frustration related to depressive symptoms among youth from four culturally diverse countries. Heissel et al. [10] found that, specifically, the dimension of basic psychological need frustration predicted adults’ ill-being in a heterogeneous sample with a large age range. A recent study by Pietrek et al. [11] showed that people with depressive disorder experience their basic psychological needs as less satisfied and more frustrated than people without depressive symptoms. Despite this pattern of findings, less is known about possible mechanisms in the relation between need frustration and symptoms of psychopathology.

According to the Response Styles Theory (RST) [13], rumination is a cognitive process that involves the repetitive analysis of oneself and one’s problems, concerns, feelings of distress and depressed mood [13,14]. Rumination has consistently been found to be a robust predictor of depression [14,15,16,17]. Importantly, in non-clinical samples, rumination predicts higher levels of depressive symptoms and is a risk factor for the onset of Major Depressive Disorder (MDD) [16,17,18]. In individuals with clinical depression, the tendency to ruminate seems to be relatively stable, although patients experience significant changes in mood and symptom severity [19,20,21]. In the current literature, rumination is understood as a trait-like transdiagnostic risk factor that precedes depressive disorders, as well as many other disorders [22,23,24]. Central to the Response Styles Theory is the assumption that rumination is a habit-like response triggered by low mood [17].

Referring to the most widely used measurement tool for assessing rumination, namely the Ruminative Response Scale (RRS), accumulating research indicates the importance of differentiating between two aspects of rumination, namely reflection and brooding [25,26]. The first factor, reflection, is defined as “purposeful turning inward to engage in cognitive problem solving to alleviate one’s depressive symptoms” [26]. The second factor, brooding, reflects “a passive comparison of one’s current situation with some unachieved standard” [26]. On closer inspection of the Response Styles Scale Items the reflection-items describe the attempt to analyze and understand the inner experience (e.g., “go away by yourself and think about why you feel this way”; “write down what you are thinking about and analyze it”), while the brooding items additionally specify the content of this process, which is characterized by self-reproach (e.g., “Think ‘why do I always react this way?’”), penalty experience (e.g., “Think ‘What am I doing to deserve this?’”), and helplessness experience (e.g., “Think about a recent situation, wishing it had gone better”). In line with this, only brooding was found to predict less satisfaction in relationships [27] and to correlate with impaired cognitive functioning [28], reduced autobiographical memory specificity [29], negative cognitive styles [30], and suicidal ideation as well as self-criticism [31]. Repeatedly, a stronger relation of brooding with depressive symptoms was identified when compared to reflection [21,32,33,34]. In further research, rumination has been found to be consistently associated with increased negativity orientation and negative coping styles in both healthy and depressed individuals [30,35,36,37], whereas reflective rumination has a less clear association with negative outcomes and, quite the opposite, has been repeatedly associated with beneficial processes (e.g., mindfulness, active coping strategies) [38,39,40]. This is gathered in a recent article by Satyshur et al. [41]; who examined the neural basis of the two processes in their study.

Although no study has directly addressed the potential underlying mechanism between basic psychological need frustration and depressive symptoms to date, related research has demonstrated the mediating effect of rumination between individual factors such as self-criticism [42], neuroticism [43], mindfulness [44], autobiographical memory [45], and self-compassion [46] with depression. Accordingly, the mediating effect of rumination between individual factors and the presence of depressive symptoms is evident. In terms of basic psychological needs, Luyckx et al. [47] found that satisfaction of the basic psychological needs was negatively associated with ruminative exploration (i.e., an inner process of analysis that is characterized by inhibition, indecision and poor positioning) in a sample of high school and college students. Basic psychological need frustration was not measured. However, a study by van der Kaap-Deeder [48] showed that depressive symptoms related positively to rumination over need-frustrating memories. Related research has also found that certain developmental circumstances and socialization processes (such as over-controlling parenting and reduced positive reinforcement) can lead to habitual rumination [49]. For example, ongoing stress and a reduced sense of mastery predicted increases in rumination over time [50]. Undergraduates who reported over-controlling parenting during their childhood also reported higher rumination [42]. In an experimental study it was found that individuals high in self-critical perfectionism reacted more often to a competence-frustrating experience with rumination [7]. Taken together, these research findings suggest that people whose basic psychological needs are thwarted are more prone to develop maladaptive coping strategies, such as rumination, and that subsequent need-frustrating experiences and associated emotional states (e.g., dysphoric mood) trigger these strategies.

In addition to this indirect empirical support, there are also theoretical considerations in what way basic need frustration may be associated with rumination. According to SDT alongside symptom costs, coping strategies can be used to deal with experienced need frustration. Vansteenkiste et al. [2] distinguish between (a) compensatory behavior [51,52,53,54,55] and (b) need substitutes [56,57,58]. Compensatory behavior can involve rigid behavior patterns (e.g., compulsive acts). Need substitutes are defined as goals that people engage in to compensate for experienced need frustration, for instance striving for materialism or a perfect body [1]. Such mechanisms may possibly be healthier or less healthy, of short or sustained duration, but are all aimed at dealing with need frustration and thus prevent a greater impairment of health. Therefore, compensatory attempts to cope with need frustration can take various forms at a behavioral level but may also work on the level of cognitive processes, i.e., within our thoughts and ideas. Specifically, rumination may be understood as an attempt to control the unpleasant feelings triggered by need frustration by analyzing and trying to understand them. However, the brooding type of rumination seems to be characterized by self-reproach and stagnation. In summary, although experienced need frustration can directly engender mental health costs, it can also set in motion compensatory strategies that prove more or less successful in averting psychopathological symptoms such as rumination.

The present work makes a novel contribution by relating BPNT to RST, an established theory of emotion regulation processing, furthering our understanding of the roles of need frustration and habitual rumination in relation to depressive symptoms. The specific aim of this study was to investigate the possible associations between basic need frustration to depressive symptoms and rumination. We hypothesized brooding and basic need frustration would have the strongest positive correlations relative to reflective rumination. We further predicted that brooding would mediate the association between basic need frustration and depressive symptoms.

## 2. Method

### 2.1. Participants

The current data were based on a sample consisting of N = 221 participants (55.2% female) with a mean age of 27.95 years (range = 18–62, SD = 10.51) from a larger questionnaire-based survey [10]. Different recruitment contexts were targeted to achieve a less selective sample. Specifically, 124 university students were recruited from the University of Potsdam, and 97 working adults were recruited from the broader community in Berlin. Besides the minimum age of 18, there were no further inclusion or exclusion criteria. Participants were approached and recruited by study assessors on university campus or other life contexts. Questionnaires were handed out in hardcopy format by four assessors. Table 1 summarizes demographics of the examined sample. Ethical approval for this study was obtained from the Ethics Committee of Potsdam University (No. 41/2015). Following written consent, participants completed the following set of self-report measures.

### 2.2. Measures

#### 2.2.1. Basic Psychological Need Frustration

To assess basic psychological need frustration, we used the need frustration scale of the German version of the Basic Psychological Need Satisfaction and Frustration Scale (BPNSFS) [9,10]. This subscale consists of three subscales (with four items each) that relate to the three basic needs for autonomy, relatedness, and competence. Items were rated on a 5-point Likert scale, ranging from 1 (completely disagree) to 5 (completely agree), with higher scores indicating greater need frustration respectively. The internal consistency for each scale proved to be satisfactory, with Cronbach’s alpha 0.81 for autonomy frustration, 0.73 for relatedness frustration and 0.70 for competence frustration.

#### 2.2.2. Rumination

The RSQ-10D from Huffziger and Kühner [59] was adapted from the original Ruminative Response Scale (RRS) [26]. The RSQ-10D is a self-report questionnaire, which includes 10 items describing brooding and reflection (with five questions each) as possible responses to depressed mood. Items are rated on a 4-point Likert-Scale, ranging from 1 (almost never) to 4 (almost always). A total score can be calculated by summing all 10 items. Internal consistency has been documented to be acceptable [59], with Cronbach’s alphas in three subsamples for Brooding ranging between 0.60 and 0.75 and for Reflection between 0.56 and 0.75. Cronbach’s alphas in the present sample were adequate with 0.76 for the RRS total scale and 0.69 for both the brooding and the reflection subscale.

#### 2.2.3. Depressive Symptoms

Depressive symptoms were measured with the 15-item CES-D scale [60,61]. The CES-D asks about the frequency of diverse depressive symptoms experienced in the last week, assessed on a 4-point scale ranging from 0 (rarely or none of the time [less than 1 day]) to 3 (most or all the time [5–7 days]). Scores range from 0 to 45, with higher scores indicating higher levels of depressive symptoms. Summed scores of 17 and above indicate clinically relevant symptoms. Cronbach´s alpha of 0.87 was obtained in the present study.

### 2.3. Data Analysis

All data was processed using R version 4.2.0. In a first step, associations between basic psychological need frustration, rumination, and depressive symptoms were investigated via correlational analyses. In a second step, specific associations as derived from the discussed theoretical perspectives were tested in a multiple mediation model. More precisely, indirect pathways via two facets of rumination (brooding and reflection) were added, mediating the association of need frustration and depressive symptoms. Referring to the simulations of [62] and assuming small to moderate effect size for a-path and b-path, a sample size of n = 148 would be needed using bias corrected bootstrap method. Mediation analysis was performed with the “sem”-function of the lavaan package [63]. To account for the observed non-normality of the errors bootstrapping method (for 5000 samples) was used to estimate standard errors. In addition to *p*-values asymmetric confidence intervals with bootstrapping method were used [64] and provided to verify the effect for significance. When zero was not included in the 95% confidence interval, the effect was considered significant. A subsequent mediation model was formulated to consider gender as a covariate. To facilitate interpretation, we report standardized estimates and confidence intervals.

## 3. Results

Correlations between need frustration, rumination, and depressive symptoms confirmed predicted directions. The relation between rumination and need frustration was moderately statistically significant. The brooding facet of rumination was found to correlate even stronger with both need frustration and depressive symptoms. The association between need frustration and depressive symptoms was also found to be very strong. Correlations of the reflection subscale with need frustration constructs and depressive symptoms were partly not significant and all were lower when compared to the brooding subscale. Table 2 summarizes means, standard deviations and correlations among the study variables.

**Figure 1:**
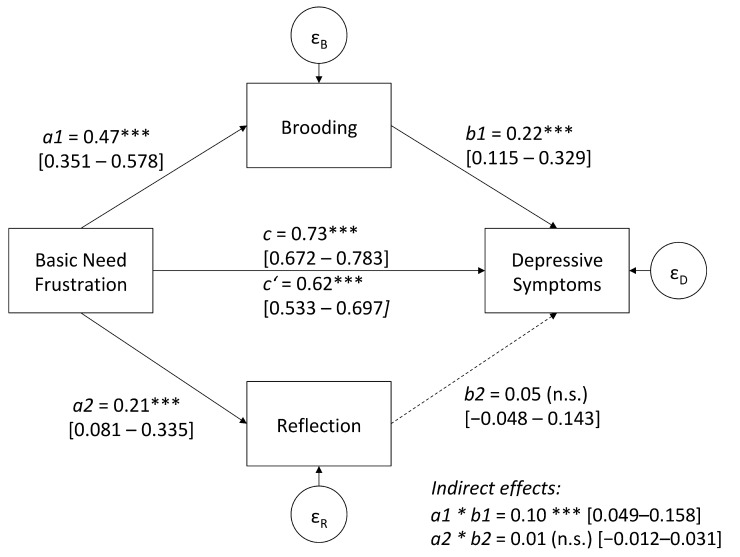
Multiple mediation model; standardized 95% confidence intervals are presented in square brackets; *** *p* < 0.001.

Structural Path Analyses revealed that brooding partially mediated the association between need frustration and depressive symptoms, whereas reflection was not found to mediate this relation. The true indirect effect via brooding was calculated to lie between CI_a1*b1_ = 0.049–0.158, respectively. Because zero is not included in the 95% confidence interval, it can be concluded that the indirect effect is significantly different from zero at *p* < 0.05. The direct effect from need frustration to depressive symptoms remained significant (c’ = 0.59, *p* < 0.05) despite the indirect pathway via rumination. The explained variance of depressive symptoms was R^2^ = 0.58, for brooding it was R^2^ = 0.22, and for Reflection R^2^ = 0.04. Figure 1 displays the multiple mediation model with parameters and confidence intervals. Including gender as a covariate, the same pattern of findings emerged showing a partial mediation effect via the brooding facet of rumination (CI_a1*b1_ = 0.047–0.155). In addition, gender had a significant direct effect on brooding and reflection but not on the extent of depressive symptoms.

## 4. Discussion

This is the first study examining underlying mechanisms in the strong correlation of basic psychological need frustration and depressive symptoms by relating Basic Psychological Needs Theory and Response Styles Theory. As theoretically and empirically derived, rumination was tested as a trait-like mediator between need frustration and depressive symptoms. Findings showed that the relation between psychological need frustration and depressive symptoms is partially mediated by brooding, thus supporting assumptions of SDT. Vansteenkiste and Ryan [5] propose need-thwarting environments to promote maladaptive coping strategies, which can become habitual. The present study revealed that individuals who experience their basic psychological needs as frustrated tend to show habitual rumination. At the same time, the association between need frustration and depressive symptoms was still significant and strong after introducing the mediator variables. Indeed, need frustration has been shown to be associated with both direct costs to psychological well-being and indirect costs through compensatory mechanisms. Complementary to this, rumination is likely to be only one among diverse compensatory attempts to handle basic need frustration that were not examined here.

Furthermore, in line with previous studies, there is a stronger association between depressive symptoms and brooding compared to the reflection component of rumination [21,32,33,34]. This leads previous studies to conclude that brooding, relative to reflection, represents the particularly maladaptive component of rumination [33,41], whereas the reflection component correlates with basic need frustration, but does not predict depressive symptoms. Rumination can thus be understood as a coping attempt in dealing with experienced need frustration, which is not dysfunctional per se. Rather, this seems to depend on the respective concrete processing mode. Specifically, it is the brooding mode that coincides with the occurrence of depressive symptoms. This view is consistent with research on rumination from the perspective of Control Theory [24,49,65], which does present rumination as a potentially successful strategy for dealing with goal discrepancies [14].

In this cross-sectional study, individual factors that possibly act as vulnerability factors for the onset of a depressive disorder were identified. Additionally, this study contributes to employ BPNT as a conceptual framework, to better understand environmental factors in the development of maladaptive mental habits, specifically, rumination. Thus, BPNT could complement integrative models of the development of rumination [24,66] by understanding and naming environmental stressors as need-frustrating environments in which balanced satisfaction of the three basic psychological needs has not been achieved [11].

### Limitations

We acknowledge that the results also need to be interpreted in relation to some methodological limitations. First, the findings are based on cross sectional data using a modest sized non-clinical sample, and hence, causality cannot be determined between variables examined. However, the theoretical underpinnings of this study are based on the proposition that psychological need frustration is a developmental antecedent of rumination. Although this is reasonable from an ontogenetic perspective, the reciprocity between these variables is possible. Second, the transversal nature of constructs examined is a further limitation. Concerning the BPNSFS we assume that both, enduring and situational environmental experiences are reflected in self-report. This is critical because it can be argued that increased depressive symptoms are associated with a negative cognitive bias that colors self-report in terms of experienced need satisfaction and frustration. Third, the results are based on self-report survey data, thereby raising the issue of common method variance. Finally, the current findings cannot be generalized to clinical populations, as scores on depressive symptoms were overall low. Future research is needed to examine if rumination, particularly brooding (relative to reflective rumination) is a mediator predicting the onset and maintenance of MDD based on thwarted psychological needs using a longitudinal design and with a larger sized sample comprising individuals with moderate to severe levels of depression.

A content-related limitation was the sole focus on rumination as a possible mediator. More research is needed examining multiple relevant mediators in the relation between need frustration and depressive symptoms, such as emotion regulation (e.g., suppression) and motivation (amotivation/helplessness). By doing so, future research could provide a more comprehensive model on the mechanisms in depression.

Further, given the cross-sectional design, the present study could not examine more in-depth theoretical assumptions outlined in the introduction. Notably, BPNT focuses on the role of the social environment supporting or thwarting three essential basic psychological needs. This refers to both past environments (that lead to the formation of the mechanism) and current environments (that maintain and trigger the mechanism). However, potential causative environmental aspects were not directly assessed in this study; hence, this warrants further investigation in future longitudinal study designs.

## 5. Conclusions

This study demonstrated the potential of including additional influencing factors to identify mediating mechanisms (e.g., brooding) between need frustration and depressive symptoms, which are strongly interrelated. In the present nonclinical sample, individuals who experienced their basic psychological needs frustrated showed depressive symptoms and tended to brood. This may place them at heightened risk for developing psychopathology (i.e., MDD). Therefore, the current findings support the usefulness of preventive interventions targeting emotion regulation strategies to manage rumination tendencies alongside reducing psychological frustration needs, and to reduce the occurrence of mental health disorders.

## Figures and Tables

**Table 1:** Demographic characteristics of the examined sample.

	Total	University Students	Working Adults
N	221	124	97
Gender			
Male (%)	99 (44.8)	61 (49.2)	38 (39.2)
Female (%)	122 (55.2)	63 (50.8)	59 (60.8)
Age			
Range (years)	18–62	18–37	18–62
Mean (years)	27.95	22.57	34.78
*SD* (years)	10.51	3.07	12.49
University entrance qualification (%)	201 (90.9)	124 (100)	77 (79.4)
Higher education (%)	76 (34.4)	30 (24.1) ^a^	46 (47.4)
Income (%)			
<1.000 €	142 (64.3)	111 (89.3)	31 (32.3)
1.000–3.000 €	48 (21.71)	10 (8.2)	38 (39.6)
>3.000 €	30 (13.6)	3 (2.5)	27 (28.1)

Note. ^a^ 58.9% of the university students specified that they were currently obtaining their education.

**Table 2:** Descriptive statistics and correlations for study measures and subscales (N = 221).

Measure	M	SD	1	2	3	4	5	6	7
1. Rumination	19.99	5.13	-						
2. Brooding	10.05	2.95	0.83 **	-					
3. Reflection	9.90	3.14	0.85 **	0.42 **	-				
4. Need Frustration	23.05	7.00	0.40 **	0.47 **	0.21 *	-			
5. Autonomy	9.70	3.21	0.36 **	0.38 **	0.22 *	0.83 **	-		
6. Competence	7.34	2.82	0.37 **	0.43 **	0.20 *	0.84 **	0.53 **	-	
7. Relatedness	6.05	2.55	0.24 **	0.33 **	0.08	0.78 **	0.44 **	0.53 **	-
8. Depressive Symptoms	9.64	6.84	0.47 **	0.53 **	0.27 **	0.78 **	0.57 **	0.64 **	0.57 **

Note. Rumination was assessed with the RRS consisting of two subscales (brooding and reflection); basic psychological need frustration was measured via the BPNSFS including three subscales (autonomy, competence, and relatedness); depressive symptoms were assessed using the CES-D. * *p* < 0.05; ** *p* < 0.01; probability is “holm” adjusted for multiple tests.

## Data Availability

The data supporting the findings of this study are available from the corresponding author upon reasonable request.
